# Ly49G, but not Ly49C/I, is dispensable for diverse antigen-specific memory NK cell responses in H-2^d^ and H-2^b^ mice

**DOI:** 10.1093/jimmun/vkaf105

**Published:** 2025-05-17

**Authors:** Gayani S Gamage, Daniel Medina-Luna, Michal Scur, Haggag S Zein, Sayanti Dey, Safyha Bryan, Andrew Wight, Zhongjun Dong, Brendon D Parsons, Mir Munir A Rahim, Andrew P Makrigiannis

**Affiliations:** Department of Microbiology and Immunology, Dalhousie University, Halifax, NS, Canada; Department of Microbiology and Immunology, Dalhousie University, Halifax, NS, Canada; Beatrice Hunter Cancer Research Institute, Dalhousie University, Halifax, NS, Canada; Department of Microbiology and Immunology, Dalhousie University, Halifax, NS, Canada; Department of Microbiology and Immunology, Dalhousie University, Halifax, NS, Canada; Department of Microbiology and Immunology, Dalhousie University, Halifax, NS, Canada; Department of Microbiology and Immunology, Dalhousie University, Halifax, NS, Canada; Department of Immunology, Dana-Farber Cancer Institute, Boston, MA, United States; Beijing Key Lab for Immunological Research on Chronic Diseases, School of Medicine and Institute for Immunology, Tsinghua University, Beijing, China; Department of Laboratory Medicine & Pathology, University of Alberta, Edmonton, AB, Canada; Department of Biomedical Sciences, University of Windsor, Windsor, ON, Canada; Department of Microbiology and Immunology, Dalhousie University, Halifax, NS, Canada; Beatrice Hunter Cancer Research Institute, Dalhousie University, Halifax, NS, Canada

**Keywords:** class-I MHC, immune memory, Ly49, natural killer cell

## Abstract

Immunological memory is a hallmark of the adaptive immune system. However, considerable evidence indicates that the natural killer (NK) cell subset of innate lymphoid cells also mediates specific memory responses to diverse antigens, including peptides. Even though the existence of NK cell memory is established, the mechanism behind NK cell adaptive responses is yet to be elucidated. Previously, we observed that the Ly49 family of class-I MHC receptors in mice are critical for the formation of adaptive NK cell memory responses. To define the nature of Ly49 involvement in NK cell memory responses, we investigated the contribution of individual Ly49 receptors and their defined class-I MHC ligands. We determined that the Ly49 requirement for the generation of NK memory responses is not uniform. Specifically, Ly49C and/or Ly49I proteins are indispensable for the adaptive NK cell responses as assessed by contact hypersensitivity recall responses to haptens and peptides, in H-2^b^ and H-2^d^ MHC backgrounds. In contrast, the highly expressed inhibitory receptor, Ly49G, did not appear to play any role in NK cell memory responses as determined using antibody-mediated subset depletion and gene-deficient mouse models, even in strains containing known ligands for Ly49G. These findings point to a unique role for Ly49C/I in adaptive NK cell antigen recognition and provide further insight into the mechanism behind adaptive NK cell responses.

## Introduction

The ability to generate long-lived, diverse antigen-specific recall responses upon re-exposure to the same pathogen is considered a feature unique to T and B cells of the conventional adaptive immune system. However, it is now clear that natural killer cells (NK cells), classically defined as cells of the innate immune system, also possess the potential for immunological memory.^1^ Conventional NK cells are cytotoxic lymphocytes that can spontaneously kill stressed, and virus-infected cells as well as cells undergoing tumorigenesis. Similar to cytotoxic CD8^+^ T lymphocytes, NK cells lyse tumour or virus-infected cells by releasing cytotoxic granules and secreting a variety of inflammatory cytokines and chemokines.^2^^,^^3^ However, unlike T and B lymphocytes, which express antigen-specific receptors as a result of gene-rearrangement, NK cells are equipped with a broad array of germline-encoded activating and inhibitory receptors, which they use to sense their environment to discriminate cellular signatures of self from non-self or altered-self.^4^ The loss or decrease in the surface expression of ligand proteins on tumour cells or virally infected cells for inhibitory NK cell receptors, along with the detection of ligands of activating receptors, prompts NK cells to become activated and deploy their effector functions. NK cell activation due to the loss or downregulation of a self-ligand for an inhibitory receptor is known as “missing-self” response.^5^^,^^6^ The subsequent discovery that the interaction between inhibitory killer cell immunoglobulin receptors (KIRs) in humans or Ly49 in mice with class I major histocompatibility complex (MHC-I) molecules drives NK cell tolerance to healthy normal cells revealed the molecular mechanism behind ‘missing-self’ responses, which was confirmed in Ly49-deficient mice.^7^^,^^8^

Due to the lack of known antigen-specific receptors, NK cells have always been regarded as short-lived cytolytic cells that rapidly respond against pathogens in an antigen-independent manner. However, challenging this paradigm, mounting evidence in both mice and humans has shown that NK cells can generate long-lived, antigen-specific memory responses similar to T and B cells.^9^^,^^10^ Currently, the immune memory properties of NK cells have been described in three different models: cytomegalovirus (CMV)-reactive NK cell memory,^11^^,^^12^ cytokine-induced NK cell memory^13^^,^^14^ and adaptive-hepatic NK cell memory.^15^^,^^16^ Among these 3 models, adaptive hepatic NK cell memory is distinct from the other 2 models as hepatic memory NK cells can generate antigen-specific memory responses against a broad range of antigens, whereas the formation of NK cell memory in CMV and cytokine-induced memory is limited to highly specific ligand/receptor interaction and strictly defined cytokines, respectively.^17^ However, the mechanism behind how hepatic adaptive NK cells acquire antigen-specificity in the absence of somatically rearranged receptors is still unclear. Hepatic memory NK cells are reported to express markers such as Thy1, NK1.1, NKp46, NKG2D, CXCR6, IL-7Ra, CD49a, and Ly49C or Ly49I.^15^^,^^16^^,^^18^ Ly49C and Ly49I are closely related, likely as a result of recent gene duplication, and are detected by a single mAb, 5E6, that binds both proteins and will be referred to hereafter as Ly49C/I.^19^ Unfortunately, none of these markers explain how NK cells specifically recognize antigens for which there are likely no germline-encoded receptors.

The discovery of Ly49C and/or Ly49I as markers in adaptive hepatic NK cell memory led us to explore whether Ly49s are somehow involved in antigen recognition by memory NK cells. The Ly49s are type II C-type lectin-like glycoproteins encoded by a highly polygenic and polymorphic gene family designated as *Klra.*^20^ The members of the Ly49 family comprise both DAP12-associating “activating” and ITIM-containing ‘inhibitory’ receptors, which can recognize MHC-I molecules and MHC-I-like proteins in normal as well as altered cells.^21^^,^^22^ Thus, inhibitory Ly49 proteins play a critical role in NK cell “missing-self” response by preventing NK cell activation against a target cell with normal levels of MHC-I proteins. In addition to regulating NK cell functions, Ly49 proteins are also involved in NK cell education, where NK cells acquire functional competency by successfully engaging a self-ligand with an inhibitory receptor during their development.^23^^,^^24^

A novel role of Ly49s was brought to light when we discovered that Ly49s are critical for the formation of adaptive hepatic NK cell responses.^25^^,^^26^ Specifically, we found that expression and ligand-binding of inhibitory receptors, Ly49C and/or Ly49I, are essential for adaptive NK cell responses during both antigen-sensitization and recall/challenge phases and that these receptors drive NK cell memory formation towards diverse haptens and peptides by interacting with their cognate ligand, MHC-I. Furthermore, we discovered that Ly49-interactive residues of the presented peptide antigens dictate the antigen specificity of the NK cell memory responses, thus raising the possibility that Ly49C and/or Ly49I are directly involved in the antigen recognition behind NK cell memory.^25^^,^^26^

However, as the Ly49 gene family consists of many other members in addition to Ly49C and Ly49I, it raises the question of whether other inhibitory Ly49 possess parallel roles in NK cell memory responses or whether NK cell memory is an intrinsic feature of Ly49C/I. Here, we investigated the role of the inhibitory receptor Ly49G in adaptive NK cell responses, using Ly49-deficient mice and Ly49 subset depletion via mAb injection. Like Ly49C/I, Ly49G is expressed on a large subset of NK cells and is conserved in all known inbred mouse strains.^27^ Using contact hypersensitivity ear-swelling assays in *Rag1^−/−^*H-2^d^ mice, which express the known MHC-I ligands for Ly49G, we found that Ly49G is not involved in mediating NK cell memory responses. However, Ly49C/I mediate NK cell memory responses by interacting with class-I MHC molecules of the H-2^d^ haplotype, thereby indicating that NK cell memory formation and antigen-specificity may be intrinsic to select Ly49 receptors, such as Ly49C/I, regardless of MHC background.

## Methods and materials

### Mice

B6.129S7-*Rag1*^tm1Mom^*/*J(*Rag1^−/−^*), B6.C-*H2^d^*^/^bByJ (H-2^d^), and C57BL/6N-Klra7^em1(IMPC)J^/J (Ly49G^*−/−*^) mice on a C57BL/6 background were purchased from the Jackson laboratory. The generation of B6-*Klra3^tm1^Klra9^tm1^* (Ly49C/I^*−/−*^) mice was previously described.^8^  *Rag1^−/−^* mice were crossed with H-2^d^ and Ly49C/I^*−/−*^ mice to produce *Rag1^−/−^*H-2^d^, and *Rag1^−/−^* Ly49C/I^*−/−*^ mice, respectively. *Rag1^−/−^*Ly49G^*−/−*^H-2^d^ mice were produced by crossing *Rag1^−/−^*H-2^d^ mice with Ly49G^*−/−*^ mice. To obtain *Rag1^−/−^*Ly49C/I^*−/−*^H-2^d^ mice, *Rag1^−/−^*H-2^d^ mice were crossed with *Rag1^−/−^*Ly49C/I^*−/−*^ mice. Mouse genotypes were confirmed by performing PCR and flow cytometry. The primers used for mouse genotyping are listed in Table 1. All *in vivo* experiments were initiated in mice between 6 and 9 wk of age. Both female and male mice were included in each experiment. All mice were maintained in a specific pathogen-free environment, and all breeding was performed in-house. Breeding and manipulations performed on animals were conducted following the standards and protocols approved by the Dalhousie University Committee on Laboratory Animals.

**Table 1.** Primers for genomic PCR.

Primer Name	Primer Pair	Sequence
Klra3 (Ly49C)	For	ATTGAAATCCCCATTTTACCAACG
	Rev	GTCCTTGTACATAACCAAGCCCT
Klra9 (Ly49I)	For	CTTCTTGTACTCCCACGATGAA
	Rev	TCCCATCCTTGTGCATAACC
*Rag*1	Rag WT For	GAGGTTCCGCTACGACTCTG
	Rag KO For	TGGATGTGGAATGTGTGCGAG
	Rag Rev	CCGGACAAGTTTTTTCATCGT
Klra7 (Ly49G)	Ly49G Com Rev	AGCTTCTCTGGGCCTTTGAG
	Ly49GWT For	TAGTCAGACCCCACCCTTTC
	Ly49G Mut For	CATTAGCTCATTGGGGCTTC
H-2^d/b^	Eb Rev1	GTGGACACAATTCCTGTTTT
	Eb Rev2	GTGGACACAATTCCTGTTCC
	Eb For	CGACTGTAGGGCCTTAGCCTG

### Cells

YAC-1 (TIB-160; H-2^k^ lymphoma) was purchased from American Type Culture Collection (Manassas, Virginia, USA). YB2/0 (rat hybridoma) and YB.D^d^ cells were kindly provided by Dr Stephen Anderson (SAIC-Frederick, Frederick, Maryland, USA). Cells were cultured in complete RPMI medium supplemented with 10% FBS, 2 mM l-glutamine, 100 U/ml penicillin, and 100 μg/ml streptomycin.

### Contact hypersensitivity (ear-swelling) assay

The mouse ear-swelling assay was performed as previously described.^25^ Briefly, on days 0 and 1, mice were sensitized by painting the shaved abdomen skin with 50 µl of 0.5% 2,4-dinitrofluorobenzene (DNFB) (Sigma-Aldrich, # D1529-10ML) in acetone. On day 5, mice were challenged by painting the skin of their left ears with 20 μl of 0.2% DNFB in vehicle (acetone), and the right ears were painted with the vehicle alone. For peptide reactions, mice were sensitized to the indicated peptide by hock injection of 25 nM of peptide in Complete Freund’s Adjuvant (Thermo Scientific, #77140) on day 0, and boosted with the same peptide in Incomplete Freund’s Adjuvant (Thermo Scientific, #77145) on day 7. Hock injections were performed by injecting both hind legs with 50 μl emulsified peptide/adjuvant solution. Ear challenge was performed with a subcutaneous injection of 20 µl of 1 nM peptide solution in sterile 1× phosphate-buffered saline (PBS) in 1 ear and PBS alone in the other ear. Hock injections and subcutaneous injections were performed under anesthesia. The following 2 peptides were used for the peptide-induced ear-swelling assays: RGPGRAFVIT (gp160) (GenScript), an H-2D^d^ restricted peptide of the HIV gp160 envelope protein, and TYQRTRALV (TYQ) (GenScript, # RP20260), an H-2K^d^ restricted peptide of influenza NP.

### Monoclonal antibody production and purification

Anti-Ly49C/I, and anti-Ly49G monoclonal antibodies (mAb) were produced from hybridoma clones 5E6, and 4D11, respectively. The 5E6 clone was received as a gift from Dr Charles Sentman (Dartmouth Hitchcock Medical Center, Lebanon, New Hampshire, USA), and clone 4D11 was purchased from ATCC. All hybridoma clones were cultured in DMEM supplemented with 1 mM sodium pyruvate, 0.1 mM non-essential amino acids, 0.1 mM β-mercaptoethanol, 100 U/ml penicillin, and 100 μg/ml streptomycin. Culture supernatants were centrifuged (500× g for 10 min) and filtered through a 0.45 μm filter. Antibodies were then purified using Protein G Sepharose chromatography (ProteinMods, #292PGG16) and dialyzed in 1× PBS buffer (pH 7.4) overnight. Purified mAbs were concentrated in an Amicon ultra-15 centrifugal filter unit with an Ultracel-100 kDa membrane (EMD Millipore). Antibody purity was determined by SDS-PAGE and concentration by spectrophotometric measurement at 280 nm.

### Antibody depletions

Two days prior to the hapten or peptide sensitization, mice were injected with 200 μg of depleting mAb in sterile 1× PBS intraperitoneally (IP). To maintain the depletions of Ly49C/I^+^ and/or Ly49G^+^ NK cells throughout the experiments, mice were injected with 100 μg of anti-Ly49C/I and/or Ly49G mAbs every other day until the day of the challenge.

### Preparation of single-cell suspension from mouse tissues

Spleens were harvested and minced using glass microscopic slides. Cells were washed with 10 ml of 1× PBS and pelleted by centrifuging at 500 g for 5 min at 20°C. Red blood cells were lysed by resuspending the cells in 2 ml of ACK lysis buffer (0.15 M NH4Cl, 10 mM KHCO3, 0.1 mM EDTA) for 5 min on ice. ACK lysis was interrupted by the addition of 10 mL 1x PBS. Cells were washed with 1× PBS as above and were counted using a hemocytometer before antibody staining for flow cytometry. Bone marrow cells were isolated from mouse tibiae and femurs. Briefly, bones were dissected, and epiphyses were removed. Bone marrow cells were then flushed from the bone shafts using 1× PBS supplemented with 2% FBS, using a 25-gauge needle. The cell suspension was then passed through a 40 μm cell strainers (Falcon, #352340) and the flow-through was then collected and centrifuged at 500× g for 5 min at 4°C. The cell pellet was collected and treated with 2 ml of ACK lysis for 5 min on ice, terminated by addition of 1× PBS and centrifuged at 500× g for 5 min. After centrifugation, the cell pellet was collected and resuspended in 1× PBS and counted.

### In vitro stimulation assay

Total lymphocytes isolated from the spleens were incubated with indicated target cells at a 1:1 ratio or with phorbol 12-myristate 13-acetate (PMA, 10 μg/ml) and ionomycin (1 μg/ml) in the presence of anti-CD107a mAb, brefeldin A, and monensin (eBiosience) for 4 h. Cells were stained for surface markers followed by intracellular staining for IFN-γ using IC fixation and permeabilization reagents (eBioscience) following manufacturer’s instructions.

### Antibody staining and flow cytometry

The following antibodies were purchased from commercial sources as follows: eFluor 450-conjugated anti-CD45 (30-F11) (Invitrogen, #48-0451-82), APC-conjugated anti-CD45 (30F-11) (BioLegend, #103112), eFluor 450-conjugated anti-CD3 (17A2) (eBioscience, #48-0032-80), APC-conjugated anti-NK1.1 (PK136) (BioLegend, #108710), PE-Cy7-conjugated anti-NK1.1 (PK136) (BioLegend, #108714), FITC-conjugated anti-Ly49C/I (5E6) (BD Pharmingen, #553276), PE-conjugated anti-Ly49G (4D11) (Miltenyi Biotech, #130-102-287), FITC-conjugated anti-Ly49A (YE1/48.10.6) (BioLegend, #116805), FITC-conjugated anti-Ly49D (4E5) (BD Pharmingen, #555313),) PE-conjugated anti-Ly49H (3D10) (eBioscience, #12-58886-80), PE-conjugated anti-NKG2D (CX5) (eBioscience, #12-58882-81), FITC-conjugated anti-CD49b (DX5) (eBioscience, #115971-82), FITC-conjugated anti-CD94 (18d3) (BioLegend, #105507), FITC-conjugated anti-NKG2A/C/E (20d5) (eBioscience, #11-5896-85), eFluro 450-conjugated anti-NKp46 (29A1.4) (eBioscience, #48-3351-82), PE-conjugated anti-CD27 (LG.3A10) (BioLegend, #124209), PE-Cy7-conjugated anti-CD11b (M1/70) (BioLegend, #101216), FITC-conjugated anti-CD69 (H1.2F3) (eBioscience, #11-0691-82), purified anti-NKR-P1B (2D12) (Sigma Aldrich, #MABF-2115), and fixable viability dye eFluro780 (eBioscience,# 65-0865-14).

For flow cytometry analysis, approximately 1 × 10^6^ cells from spleens or bone marrows were used for antibody staining. Cells were washed in FACS buffer (1× PBS, 0.5% BSA, and 0.02% NaN3) and incubated in Fc block (anti-CD16/32) at 4°C for 10 min in dark. Cells were then stained with appropriate antibodies diluted in FACS buffer and incubated at 4°C for 45 min in the dark. After antibody incubation, cells were washed with FACS buffer and fixed in 400 μl of 1% paraformaldehyde (PFA). Samples were acquired using a BD FACS Celesta or BD LSR Fortessa SORP (BD Biosciences, Mississauga, Ontario, Canada) and analyzed on FlowJo software version 10.0.7 (FlowJo LLC).

### Statistical analysis

Statistical analysis was performed with Graph Pad Prism software (Graph Pad 9.0, San Diego, California, USA). Statistical analyses for single variables were performed using unpaired, 2-tailed Student *t* tests. Statistical analysis for in vitro stimulation assays was performed by 2-way ANOVA followed by Tukey’s or Bonferroni’s multiple means comparison method. For ear-swelling experiments, statistics were calculated by 2-way ANOVA followed by Bonferroni’s post hoc test. All data are presented as mean ± standard error. Significance was set at a *P* value of less than 0.05.

## Results

### Characterization of NK cells in Ly49G^*−/−*^H-2^d^ mice

Our previous research has shown that the interaction between Ly49C/I and MHC-I in an H-2^b^ background is critical for the formation of hapten and peptide-specific NK cell memory responses in *Rag1^−/−^* mice.^25^ In contrast, we found that Ly49G, another highly expressed inhibitory Ly49 family member, is dispensable for adaptive NK cell responses on this MHC-I background.^25^ However, evidence from previous studies shows that Ly49G strongly binds to MHC-I proteins of the H-2^d^ (H-2D^d,^ and H-2L^d^) but not H-2^b^ haplotypes.^28–30^ Thus, we hypothesized that inhibitory Ly49s required their cognate MHC-I ligands to mediate adaptive NK cell responses. To test our hypothesis, we studied the role of Ly49G in adaptive NK cell responses by using Ly49G^*−/−*^ mice that are congenic for the H-2^d^ haplotype.

**Figure 1.**
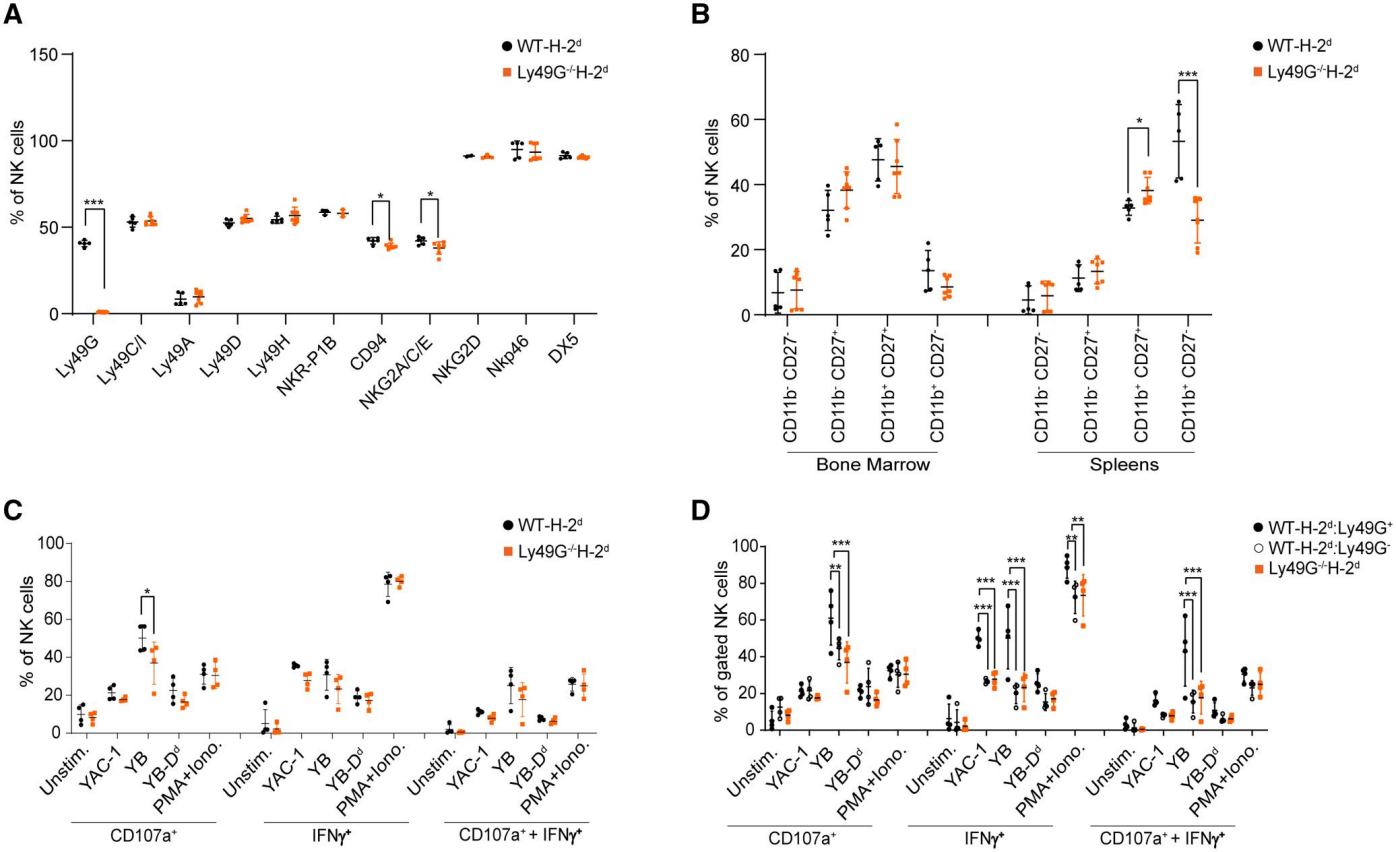
Functional characterization of NK cells from Ly49G^*−/−*^ H-2^d^ mice. (A) Flow cytometric analysis of the expression of the indicated cell surface receptors on splenic CD3^-^ NK1.1^+^ cells from WT-H-2^d^ and Ly49G^*−/−*^H-2^d^ mice. (B) Analysis of the 4 stages of the NK cell maturation according to CD11b and CD27 expression in the spleen and bone marrow from WT-H-2^d^ and Ly49G^*−/−*^ H-2^d^ mice. (C–D) In vitro stimulation of total splenic NK cells from WT and Ly49G^*−/−*^ mice on H-2^d^ background by the indicated target cells or PMA/ionomycin as a positive control. (C) and (D) represent the same pooled experiments but are analyzed as total NK cells (C) or as Ly49G^+^ or Ly49G^*−*^ subsets (D). Percentages of CD107a expression (degranulation) and intracellular IFNγ were measured in NK cells stimulated with the indicated stimuli in vitro for 4 h. Data are pooled from at least 2 independent experiments. Statistical analysis for Fig. 1B was performed using an unpaired, 2-tailed Student *t* test. For Fig. 1C, D, statistical analysis was performed using 2-way ANOVA followed by Bonferroni’s or Tukey post hoc tests, respectively. *P < 0.05; **P < 0.01; ***P < 0.001.

Flow cytometry analysis confirmed that Ly49G expression is absent in Ly49G^*−/−*^H-2^d^ mice (Fig. 1A). We then determined the expression of other Ly49 family members and other well-known NK cell receptors on splenic NK cells of WT-H-2^d^ (WT) and Ly49G^*−/−*^H-2^d^ mice. We found that the expression of Ly49C/I, Ly49A, Ly49D, Ly49H, NKp46, DX5, NKG2D, and NKR-P1B is comparable between WT and Ly49G^*−/−*^ mice. However, a slight reduction in the expression of CD94 and NK2A/D/E was detected in NK cells from Ly49G^*−/−*^ mice (Fig. 1A).

The functional maturation of NK cells is defined by the acquisition of Ly49 receptors.^31–33^ Ly49G is one of the first Ly49 expressed during the early stages of NK cell development and drives NK cell licensing by binding to MHC-I proteins in the H-2^d^ background.^29^^,^^31^^,^^34^ Thus, to explore whether Ly49G deficiency could affect NK cell maturation and differentiation in Ly49G^*−/−*^H-2^d^ mice, we assessed the expression of NK cell maturation markers, CD11b and CD27, in bone marrow (BM) and splenic NK cells from WT-H-2^d^ and Ly49G^*−/−*^H-2^d^ mice. The percentage of CD11b^+^ CD27^-^ expressing NK cells was significantly lower, and the CD11b^+^ CD27^+^ NK subset was slightly but significantly higher in spleens of the Ly49G^*−/−*^ mice, indicating that Ly49G^*−/−*^ mice have a lower percentage of terminally differentiated cytotoxic NK cells in the periphery (Fig. 1B).

As we observed changes in NK cell maturation, we next explored whether NK cell activity is affected by Ly49G deficiency. Splenic NK cells from WT and Ly49G^*−/−*^ mice were stimulated in vitro with different tumour cell targets or phorbol 12-myristate 13-acetate (PMA)/ionomycin and then assessed for the expression of CD107a and IFN-ɣ production. We found no difference in IFN-ɣ production by NK cells from Ly49G^*−/−*^ mice (Fig. 1C). However, a statistically significant decrease in CD107a release was detected in Ly49G^*−/−*^ splenic NK cells upon stimulation with YB2/0 cells (Fig. 1C). Further analysis of IFN-ɣ production and CD107a release by Ly49G^+^ and Ly49G^*−*^ NK cells from WT mice determined that Ly49G^*−*^ NK cells express significantly lower IFN-ɣ and CD107a levels upon *in vitro* stimulation. Interestingly, this is similar to the defect observed by the NK cells from Ly49G^*−/−*^ mice (Fig. 1D). Together, these results indicate that Ly49G expression is required for optimal NK cell maturation and function in the H-2^d^ MHC background.

### Ly49G expression is not required for adaptive NK cell responses in *Rag1^−/−^*H-2^d^ mice

**Figure 2:**
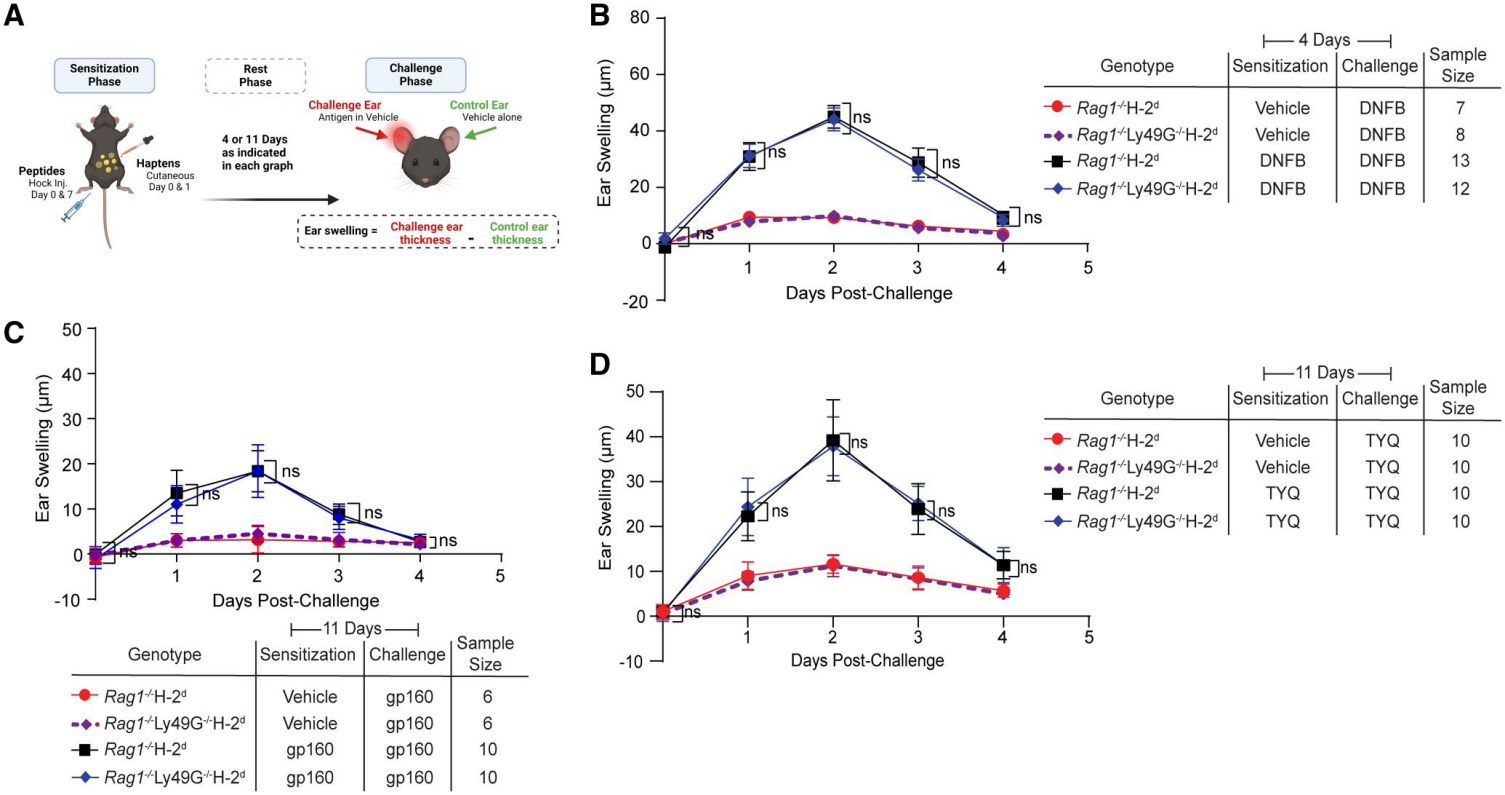
Ly49G is not required for adaptive NK cell responses in *Rag1^−/−^*H-2^d^ mice. (A) Schematic representation of hapten- and peptide-induced mouse ear swelling assay. Created in BioRender. Makrigiannis, A. (2025) https://BioRender.com/t02k134. (B) Ear swelling recall response following exposure to the chemical hapten, DNFB, in previously sensitized *Rag1^−/−^*H-2^d^ mice that lack or express Ly49G receptors. (C, D) Ear swelling responses using the peptide antigens, gp160 (RGPGRAFVTI) and TYQ (TYQRTRALV) in previously sensitized *Rag1^−/−^*H-2^d^ and *Rag1^−/−^*Ly49G^*−/−*^H-2^d^ mice. Statistical analysis was performed by two-way ANOVA, followed by the Bonferroni post hoc test, comparing all means to the means of hapten or peptide-sensitized and challenged *Rag1^−/−^*H-2^d^ mice. ns, not significant.

To determine the role of Ly49G in adaptive NK cell responses, Ly49G^*−/−*^H-2^d^ mice were crossed onto the *Rag1^−/−^* background to eliminate T and B cells. Using the mouse ear-swelling assay, we then tested the ability of these mice to form recall responses to the chemical hapten, 2,4-dinitrofluorobenzene (DNFB) (Fig. 2A). Consistent with previously published reports using *Rag1^−/−^*H-2^b^ mice,^15^^,^^16^^,^^25^ we found that *Rag1^−/−^*H-2^d^ mice display a contact hypersensitivity response to DNFB in the absence of T and B cells and this recall response was only present if mice had been previously sensitized to the DNFB (Fig. 2B). Surprisingly, mice deficient for Ly49G also formed recall responses to the hapten, and the magnitude of the ear-swelling response to DNFB was similar between Ly49G-sufficient and Ly49G-deficient *Rag1^−/−^*H-2^d^ mice (Fig. 2B). To simplify the antigen being recalled, we repeated the mouse ear-swelling assay using 2 peptide antigens, RGPGRAFVTI (gp160) and TYQRTRALV (TYQ), which are presented by H-2D^d^ and H-2K^d^ MHC-I molecules, respectively.^35^^,^^36^ The gp160 peptide complexed with an H-2D^d^ tetramer was originally used to define Ly49G ligand specificity from B6 and 129 mouse strains.^28^^,^^37^ Similar to DNFB, *Rag1^−/−^*H-2^d^ mice formed recall responses to both peptide antigens, gp160 and TYQ, in the absence of Ly49G (Fig. 2C, D). Thus, these findings suggest that Ly49G is not required for antigen-specific NK cell memory responses in *Rag1^−/−^*H-2^d^ mice.

### Ly49C/I^+^ NK cells are required for hapten and peptide-induced recall responses in *Rag1^−/−^*Ly49G^*−/−*^H-2^d^ mice

**Figure 3.**
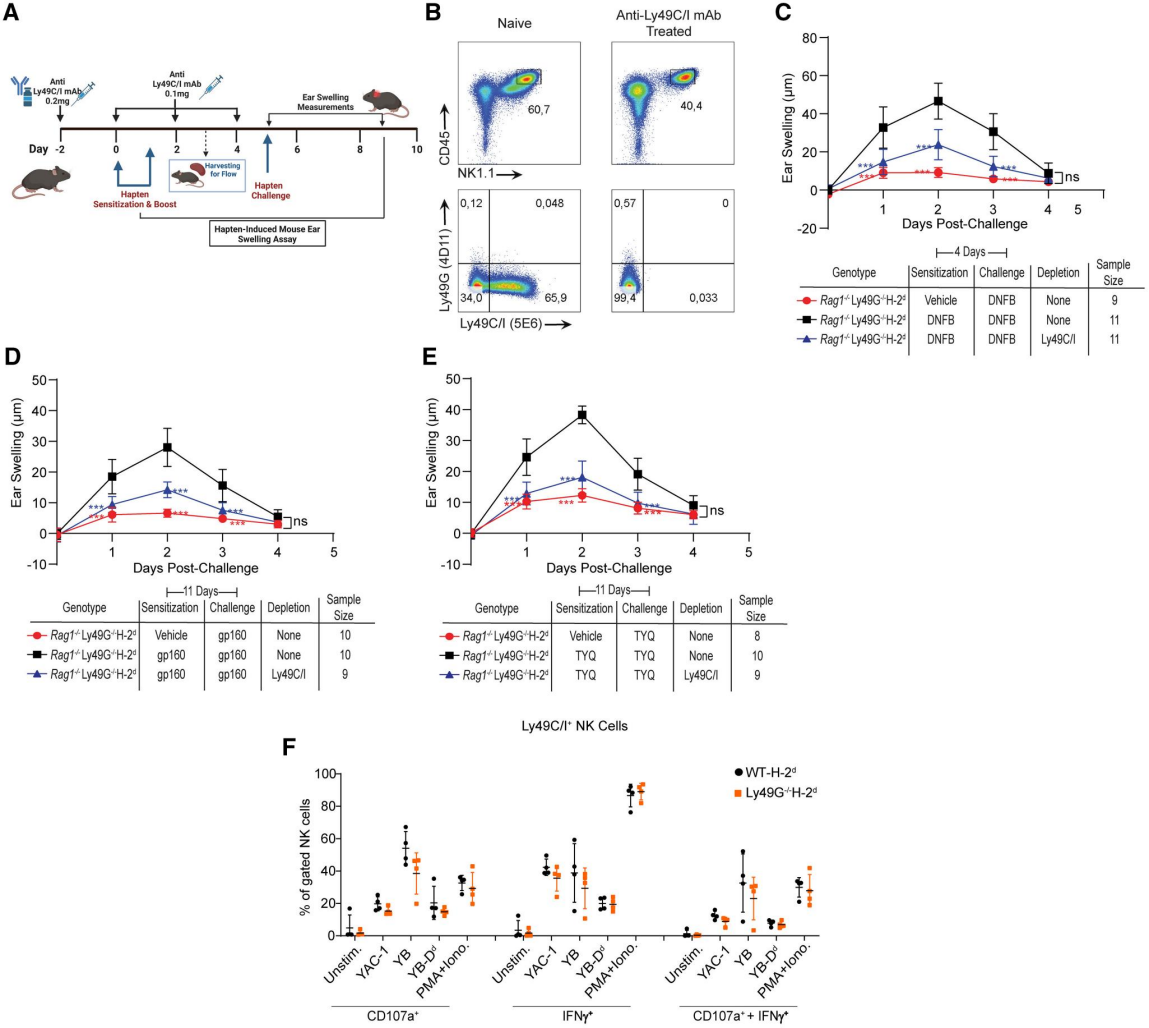
Antibody-mediated depletion of Ly49C/I^+^ NK cells significantly reduces hapten and peptide-induced recall responses in *Rag1^−/−^*Ly49G^*−/−*^H-2^d^ mice. (A) Schematic representation of hapten-induced mouse ear swelling assay following the antibody-mediated depletion of Ly49C/I^+^ NK cells from *Rag1^−/−^*Ly49G^*−/−*^H-2^d^ mice. Created in BioRender. Makrigiannis, A. (2025) https://BioRender.com/i39e862. (B) Flow cytometric analysis confirming the antibody-mediated depletion of Ly49C/I^+^ NK cells (gated on CD45^+^NK1.1^+^ cells) from spleens of *Rag1^−/−^*Ly49G^*−/−*^H-2^d^ mice following administration of 5E6 mAb. (C–E) Hapten and peptide-induced recall responses in *Rag1^−/−^*Ly49G^*−/−*^H-2^d^ mice after depletion of Ly49C/I^+^ NK cells. (F) IFN-ɣ production and degranulation by splenic Ly49C/I^+^ NK cells from WT-H-2^d^ and Ly49G^*−/−*^H-2^d^ mice following in vitro stimulation with the indicated stimuli. Statistical analysis for ear swelling was performed by 2-way ANOVA followed by the Bonferroni post hoc test comparing all means to the means of hapten or peptide-sensitized and challenged *Rag1^−/−^*H-2^d^ mice that did not receive anti-Ly49C/I mAb. Statistical analysis for *in vitro* stimulation assays was performed by 2-way ANOVA followed by Bonferroni’s multiple means comparison method. ***P < 0.001, ns, not significant.

As *Rag1^−/−^*H-2^d^ mice were able to form recall responses in the absence of Ly49G, we next determined whether the memory responses present in these mice are mediated by Ly49C and/or Ly49I, as we previously determined for H-2^b^ background mice.^25^ To investigate this, we depleted Ly49C- and Ly49I-expressing NK cells from *Rag1^−/−^*Ly49G^*−/−*^H-2^d^ by injecting anti-Ly49C/I mAb 5E6 during the sensitization phase and performed the mouse ear-swelling assay using the chemical hapten, DNFB and peptides, gp160 and TYQ (Fig. 3A). The depletion of Ly49C/I^+^ NK cells from *Rag1^−/−^*Ly49G^*−/−*^H-2^d^ mice was first confirmed by flow cytometric analysis (Fig. 3B). We then tested the ability of anti-Ly49C/I mAb-treated *Rag1^−/−^*Ly49G^*−/−*^H-2^d^ mice to form recall responses to DNFB. Interestingly, the depletion of Ly49C/I^+^ NK cells significantly reduced the DNFB-induced recall response in *Rag1^−/−^*Ly49G^*−/−*^H-2^d^ mice (Fig. 3C). Likewise, the recall responses to peptides gp160 and TYQ were significantly reduced with the depletion of Ly49C/I^+^ NK cells from *Rag1 ^−/−^*Ly49G^*−/−*^H-2^d^ mice (Fig. 3D, E). To explore whether the activity of Ly49C/I^+^ NK cells is enhanced due to the deficiency of Ly49G in our mice, we performed an *in vitro* stimulation assay to assess IFN-ɣ production and expression of CD107a by the Ly49C/I^+^ NK cells from WT-H-2^d^ and Ly49G^*−/−*^H-2^d^ mice. We found no difference in the expression of IFN-ɣ and CD107a between the Ly49C/I^+^ NK cells from WT and Ly49G^*−/−*^ mice after activation via various stimuli, suggesting the activity of Ly49C/I^+^ NK cells is not enhanced due to the deficiency of Ly49G (Fig. 3F). Collectively, these results suggest that Ly49C and/or Ly49I can mediate adaptive NK cell responses in mice of the H-2^d^ haplotype.

### Antibody-mediated depletion of Ly49C/I^+^ NK cells, but not Ly49G^+^ NK cells, eliminates memory formation to chemical haptens and peptides in *Rag1^−/−^*H-2^d^ mice

**Figure 4.**
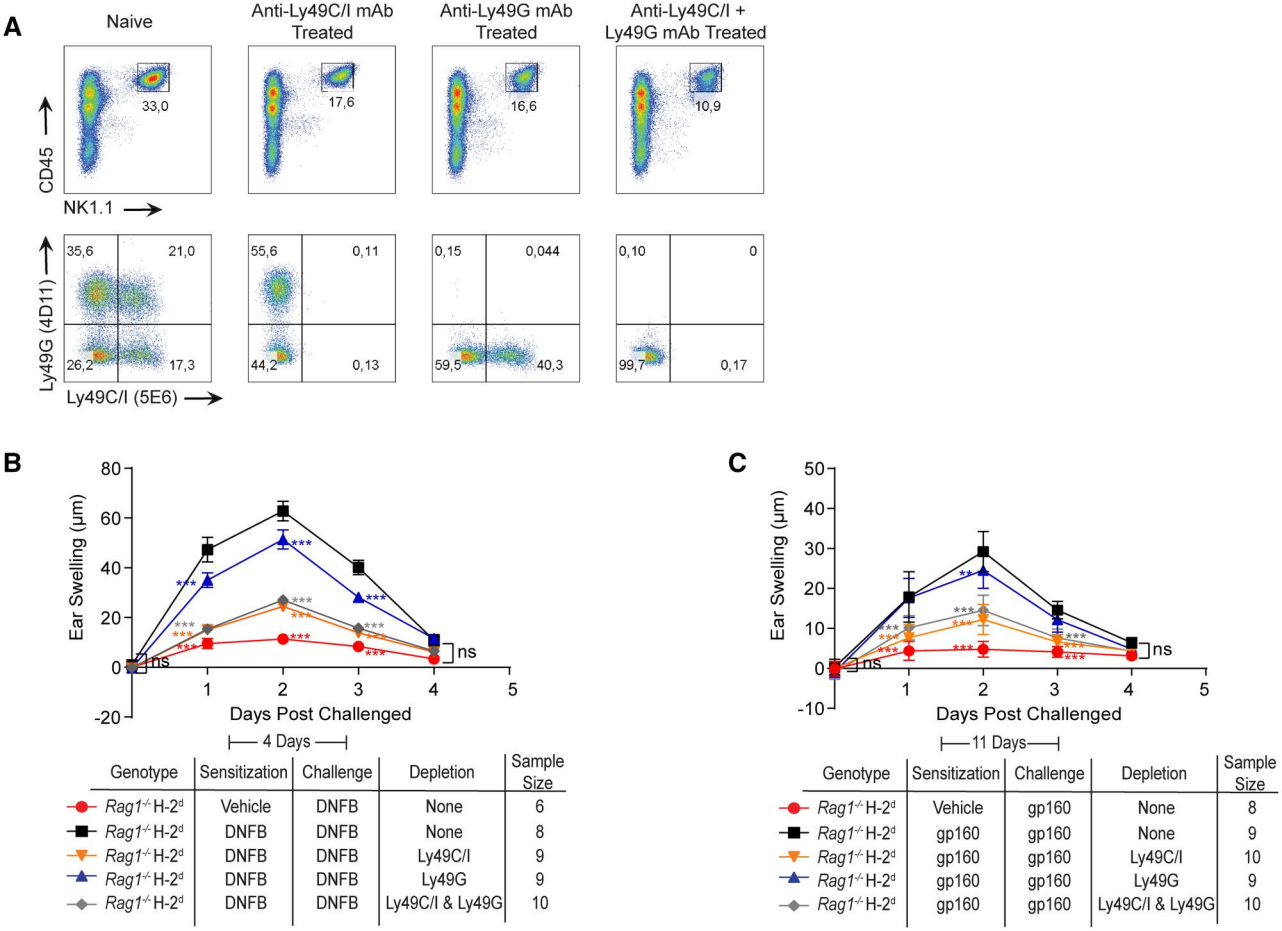
Depletion of Ly49C/I^+^ NK cells, but not Ly49G^+^ NK cells, significantly reduces memory formation to haptens and peptides in *Rag1^−/−^*H-2^d^ mice. (A) Flow analysis confirming the depletion of specific Ly49-expressing NK cell subsets (gated on CD45^+^NK1.1^+^ cells) from spleens of *Rag1^−/−^* H-2^d^ mice following the indicated antibody treatments. (B, C) Ear swelling response following hapten (B) and peptide (C) challenge in *Rag1^−/−^*H-2^d^ mice that received anti-Ly49C/I and/or Ly49G antibody treatments. Statistical analysis was performed by 2way ANOVA followed by the Bonferroni post hoc test comparing all means to the means of hapten or peptide-sensitized and challenged *Rag1^−/−^*H-2^d^ mice that did not receive antibody treatments. **P < 0.01; ***P < 0.001, ns, not significant.

To validate these results and avoid possible NK cell education defects in Ly49G^*−/−*^ mice, we repeated the DNFB and gp160-induced ear-swelling assays in *Rag1^−/−^*H-2^d^ mice by selectively depleting the NK cell populations that express Ly49C/I, Ly49G, or both Ly49C/I and Ly49G during the antigen-sensitization phase. Using flow cytometric analysis, we first confirmed antibody-mediated depletion of Ly49C/I^+^ and Ly49G^+^ NK cells from *Rag1^−/−^*H-2^d^ mice (Fig. 4A). Depletion of Ly49C/I^+^ NK cells significantly reduced memory formation to DNFB and gp160 in *Rag1^−/−^*H-2^d^, confirming our previous results (Fig. 4B, C).

Interestingly, a slight yet statistically significant reduction of the ear-swelling response to DNFB and gp160 was also observed in mice treated with depleting antibodies for Ly49G (Fig. 4B, C). However, when we compared the hapten and peptide-induced recall responses in mice that received depleting antibodies for both Ly49G and Ly49C/I, we observed that the magnitude of ear-swelling reduction in these mice is similar to the ear-swelling reduction detected by the mice that received depleting antibodies for Ly49C/I alone. Thus, these results indicate that the ear-swelling reduction observed in mice receiving depleting antibodies against Ly49G is not due to Ly49G participating in NK memory responses but is likely due to the depletion of Ly49C/I and Ly49G co-expressing NK cells (21% of total NK cells; Fig. 4A) from *Rag1^−/−^*H-2^d^ mice (Fig. 4B, C). Overall, these findings are consistent with our previous results, showing that Ly49G is not involved in mediating NK cell memory responses even in the presence of its specific MHC-I ligands. On the other hand, Ly49C and/or Ly49I is required for the induction of recall responses to chemical haptens and peptides in mice of the H-2^d^ haplotype.

### Gene disruption reveals that Ly49C and Ly49I are essential for adaptive NK cell responses in both *Rag1^−/−^*H-2^d^ and *Rag1^−/−^*H-2^b^ mice

**Figure 5.**
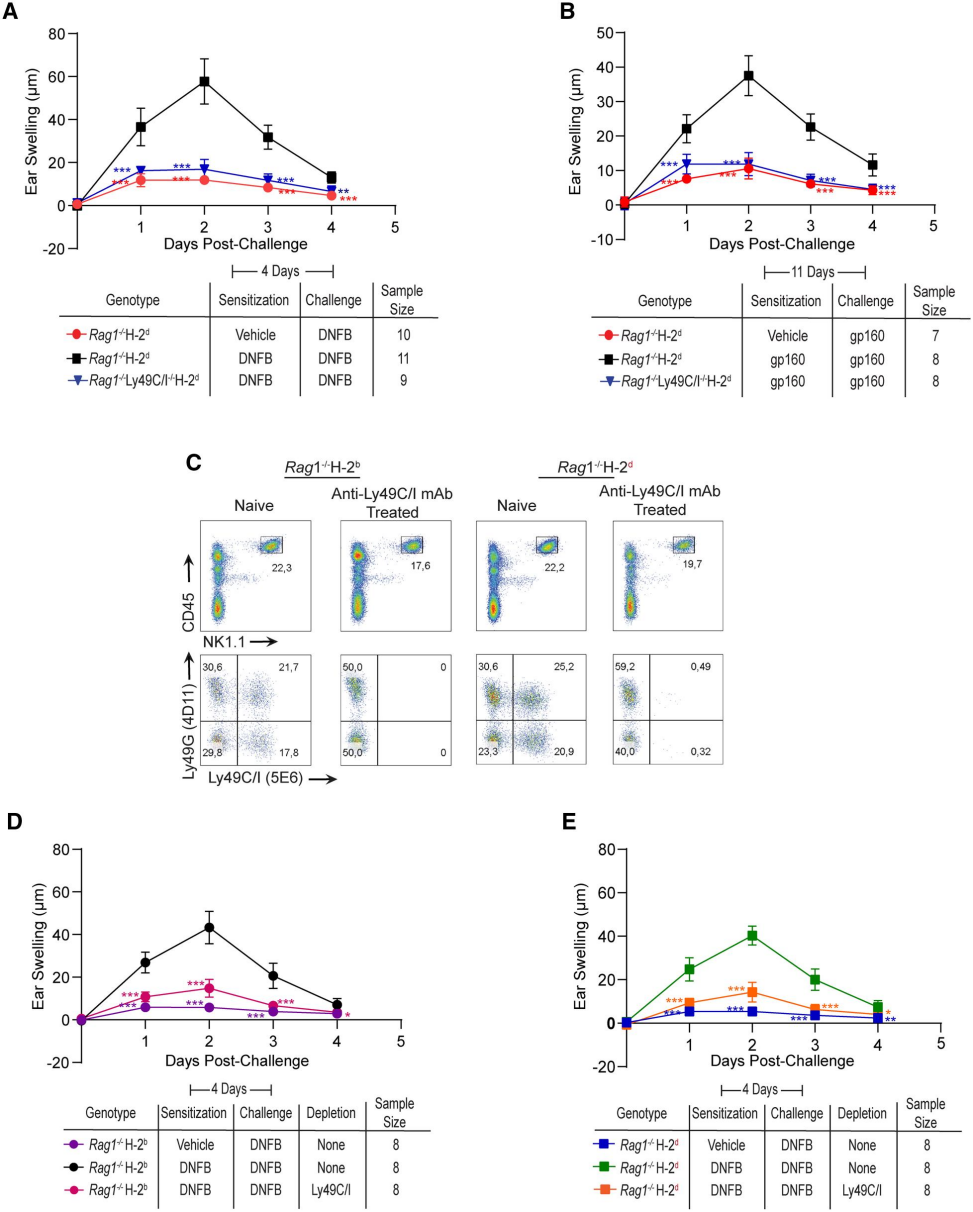
Ly49C/I expression is critical for adaptive NK cell responses in *Rag1^−/−^*H-2^d^ mice. (A, B) Hapten (A) and peptide-induced (B) ear swelling recall responses in *Rag1^−/−^*H-2^d^ mice that lack or express Ly49C and Ly49I. (C) Flow cytometry of Ly49 expression before and after antibody-mediated depletion of Ly49C/I^+^ NK cells (gated on CD45^+^NK1.1^+^ cells) from spleens of *Rag1^−/−^*H-2^b^ and *Rag1^−/−^*H-2^d^ mice. (D, E) Ear swelling responses following DNFB challenge in H-2^b^  *Rag1^−/−^* (D) or H-2^d^  *Rag1^−/−^* (E) mice after depletion of Ly49C/I^+^ NK cells. Statistical analysis for ear swelling recall experiments was performed by two-way ANOVA followed by the Bonferroni post hoc test comparing all means to the means of hapten sensitized and challenged *Rag1^−/−^*H-2^b^ or *Rag1^−/−^* H-2^d^ mice that did not receive antibody treatments. *P < 0.05; **P < 0.01*;* ***P < 0.001, ns, not significant.

To confirm our mAb depletion studies on the role of Ly49C and Ly49I in mediating adaptive NK cell responses in the H-2^d^ background, we crossed mice with disruptions in the genes encoding Ly49C and Ly49I with *Rag1^−/−^*H-2^d^. Considering our previous results, we hypothesized that genetic ablation of Ly49C and Ly49I would result in lack of memory formation to chemical haptens and peptides in *Rag1^−/−^*H-2^d^ mice. As expected, the DNFB and gp160-induced recall responses were completely abrogated in *Rag1^−/−^*Ly49C/I^*−/−*^H-2^d^ mice, thereby confirming that Ly49C and/or Ly49I functional gene presence (as opposed to Ly49G, which is still present in these animals) is essential to mediate adaptive NK cells responses in H-2^d^ MHC background (Fig. 5A, B).

As we found that Ly49C- and/or Ly49I-expressing subsets are required for adaptive NK cell responses in both H-2^b25^ and H-2^d^ MHC backgrounds (Fig. 5A, B), we then explored whether the magnitude of the Ly49C/I -driven memory responses in H-2^b^ and H-2^d^ MHC backgrounds are similar. To study this, we selectively depleted the Ly49C/I^+^ NK cells from *Rag1^−/−^*H-2^b^ and *Rag1^−/−^*H-2^d^ mice (Fig. 5C) and then performed the mouse ear-swelling assay using DNFB as antigen. Strikingly, we found that the DNFB-induced recall response is comparable between *Rag1^−/−^*H-2^b^ and *Rag1^−/−^* H-2^d^ mice (Fig. 5D, E). Similarly, we found that the depletion of Ly49C/I^+^ NK cells significantly reduced DNFB-induced recall responses in both H-2^b^ and H-2^d^ backgrounds, and this ear-swelling reduction is similar between the 2 MHC backgrounds (Fig. 5D, E). Overall, these results suggest that Ly49C and/or Ly49I can mediate adaptive NK cell responses regardless of the MHC-I molecules present in each mouse strain.

## Discussion

Many studies over the past years have elucidated that NK cells possess the ability to generate long-lasting, adaptive memory responses to a broad array of antigens.^15^^,^^16^^,^^25^^,^^38^ However, the mechanism by which NK cells mediate adaptive responses without recombinant receptors remains unknown. Previously, we reported that inhibitory receptors, Ly49C and/or Ly49I, drive adaptive NK cell responses by interacting with self-MHC-I proteins in *Rag1^−/−^* mice during both sensitization and challenge phases.^25^ To further elucidate the Ly49 receptor requirement in NK cell memory response, we explored the role of the inhibitory receptor, Ly49G, in mediating adaptive NK cell responses in the present study. Like Ly49C/I, Ly49G is found in a substantial proportion of NK cells and is conserved across all known mouse Ly49 haplotypes.^27^ In the present study, we show that Ly49G is not required for adaptive NK cell responses as genetic ablation or antibody-mediated depletion of Ly49G^+^ NK cells did not affect memory formation to chemical haptens or peptides in *Rag1^−/−^* mice that express MHC-I ligands for Ly49G. In contrast, we found that Ly49C/I, previously identified to mediate NK cell memory responses by interacting with MHC-I proteins of the H-2^b^ background, can also drive memory responses in *Rag1^−/−^* mice that are congenic for an H-2^d^ MHC-I background.

While it is interesting that Ly49C and/or Ly49I, but not Ly49G, are essential in NK cell memory responses, what makes Ly49C and Ly49I unique from other Ly49s in mediating adaptive NK cell responses remains to be uncovered. Even though we found that Ly49G is not required for NK cell memory responses, our data show that Ly49G expression is required for optimal NK cell maturation and NK cell functions in an H-2^d^ background (Fig. 1B, D). Our study is in agreement with previously published data on the role of Ly49G in NK cell licensing and effector functions in H-2^d^ mice.^29^^,^^34^^,^^39–41^ Moreover, Ly49G expression is required to mediate protection against MCMV infection in B10.D2 mice.^42^ As a whole, these studies provide evidence for the importance of Ly49G in some NK cell functions and during NK cell development. However, in the context of NK cell memory, we found that Ly49G does not seem to play any role, even in the presence of its cognate MHC-I ligands.

Conversely, we observed that Ly49C/I can induce recall responses to chemical haptens and peptides by interacting with the MHC-I proteins in the H-2^d^ background. As evident from cell adhesion and tetramer studies, Ly49C and Ly49I have a broad range of specificities and affinities towards the MHC-I ligands, many of which are overlapping.^28^^,^^43^^,^^44^ However, among these reported MHC-I ligands, Ly49C and Ly49I show the highest binding affinity towards MHC-I proteins encoded by the H-2^b^ haplotype.^28^^,^^45^ Although Ly49C/I have relatively weaker binding affinity to H-2^d^ than H-2^b^ MHC-I proteins, surprisingly, we saw no difference in the Ly49C/I-mediated recall response to DNFB between H-2^d^ vs. H-2^b^  *Rag1^−/−^* mice (Fig. 5D, E). Thus, these data suggest that Ly49-mediated NK cell memory responses are driven independently of the binding affinity of Ly49 to their MHC-I ligands. Our contact hypersensitivity experiments with Ly49G also support this, as we saw that Ly49G is dispensable for memory responses, even though Ly49G has a strong binding affinity to H-2^d^ encoded MHC-I proteins. However, further studies are required to validate whether stronger receptor-ligand affinity of various Ly49 to MHC-I ligands can mediate memory responses or whether this is an intrinsic feature of how Ly49C/I mediate antigen-adaptive NK cell memory. For example, Ly49G^BALB/c^ binds to H-2K^d^ with a higher affinity than Ly49G^B6,46^ while Ly49A^B6^ has the highest binding affinity to H-2^d^ MHC-I proteins from all the Ly49 members in this mouse strain.^47–49^

In general, Ly49 proteins do not directly interact with the peptide presented by the MHC-I complex.^28^ Instead, Ly49 engage with MHC-I underneath the peptide-binding grove formed by the heavy chain α1, α2, and α3 domains and β_2_m.^50^ Therefore, the binding of Ly49 to MHC-I molecules is driven in a peptide-dependent but not in a peptide-specific manner. However, Ly49C and Ly49I are exceptions to this rule, as these 2 receptors have been shown to engage with MHC-I in a peptide-specific manner. Hanke et al. demonstrated the first evidence for the peptide-selective nature of Ly49I, where they found that H-2K^d^ interaction with Ly49I is supported only by certain peptides.^28^ Similarly, the binding of Ly49C to H-2K^b^ is influenced by specific peptides presented by MHC-I molecules.^51^^,^^52^ Moreover, Ly49C binding to H-2K^b^ is sensitive to the anchor residue P2 and P3 positions of the peptide presented by the MHC-I complex.^53^

Based on this evidence, we previously studied whether the antigen-specificity of NK cell memory responses is driven by selective peptide residue recognition. We found that adaptive NK cell memory responses cannot differentiate between 2 different peptides that share P2 and P3 residues but are otherwise different amino acid sequences.^25^ This is consistent with a direct involvement of Ly49C and/or Ly49I in antigen recognition.^25^ However, these previous studies were performed in mice in the H-2^b^ MHC background, and the results of the present study show for the first time that Ly49C/I can mediate NK cell memory responses by interacting with MHC-I proteins of the H-2^d^ background. Thus, it is possible that Ly49C and Ly49I might employ similar mechanisms in detecting antigens regardless of the MHC-I background. Therefore, future studies focusing on how Ly49C/I interact with the peptide-bound MHC-I complex in different MHC-I haplotypes and how these interactions affect downstream signaling pathways will provide further insight into the mechanism behind the antigen-specificity of Ly49-driven NK cell memory responses.

While the involvement of Ly49C and/or Ly49I in antigen recognition is intriguing, it seems contradictory that these inhibitory receptors would activate NK cells. However, in the context of NK cell memory, Ly49C and/or Ly49I might function as activating rather than inhibitory receptors. Such paradoxical roles are not unprecedented in NK cell biology. For instance, the SLAM family receptors are well known for their ability to switch between activating and inhibitory functions depending on their interaction with different adaptor proteins, such as SAP, Ewing's sarcoma-associated transcript 2 (EAT-2) and EAT-2-related transducer (ERT).^54^ Similarly, previous research in our lab has found that Ly49Q, despite possessing an ITIM in its cytoplasmic domain, acts as a positive regulator of TLR-mediated type-I IFN production in plasmacytoid dendritic cells (pDCs).^55^^,^^56^ Furthermore, the functional competency or “licensing” of NK cells is also acquired through the binding to self-MHC-I ligands via their inhibitory receptors.^57^ Thus, it is plausible that Ly49C/I might exploit a similar molecular mechanism as NK cell licensing to mediate NK cell memory responses by achieving a higher licensing state. A recent publication by Schmied et al., which investigated the molecular mechanism behind NK cell licensing, revealed that in educated NK cells, inhibitory receptors sequester SHP-1, preventing its accumulation at the activating immune synapses formed between NK cells and target cells.^41^ They suggest that this sequestration of SHP-1 from the activating synapse reduces SHP-1's inhibitory influence on activating signals, thereby promoting NK cell activation.^41^ It would be interesting to investigate whether similar phenomena occur with Ly49C and/or Ly49I upon antigen recognition during memory formation. Ma et al. revealed that the peptide sensitivity of Ly49C is driven by subtle conformational changes in the structure of H-2K^b^ induced by different peptides through a mechanism known as dynamic allostery.^58^ They suggest that different peptides can tune the flexibility of the H-2K^b^ protein, altering the affinity of Ly49C for the H-2^b^ molecule.^58^ Thus, it is plausible that these subtle conformational changes induced by different peptides could influence the ability of Ly49C/I to sequester SHP-1 away from the activating immune synapse. Thereby reducing SHP-1's inhibitory influence on activating signals, promoting NK cell activation, and potentially facilitating memory formation. Studying whether the binding affinity of the peptide influences the magnitude of NK cell-mediated memory responses and how it affects downstream signaling molecules will provide insight into deciphering this hypothesis.

However, it is important to acknowledge that the depletion of Ly49C/I did not completely abrogate memory responses to haptens and peptides, as residual ear swelling was present even after the depletion of Ly49C/I and Ly49G-expressing NK cells (Fig. 4B, C). This implies that additional Ly49 receptors or other germline-encoded receptors may also contribute to the formation of adaptive NK cell responses in *Rag1^−/−^* mice. Recent studies have found that human NKG2C receptors play a crucial role in mediating antigen-specific NK cell memory against viral antigens through peptide recognition via HLA-E.^59^^,^^60^ This raises the possibility of the existence of a similar mechanism involving NKG2C and Qa-1 in mediating adaptive NK cell responses in our mouse model. However, if this is true, it would contradict our findings in Ly49C/I-deficient mice, where we found that the absence of these receptors leads to a complete abrogation of the hapten and peptide-induced recall responses. Therefore, an alternative explanation for the residual ear swelling observed after Ly49C/I^+^ NK cell depletion could be attributed to the emergence of new Ly49C/I^+^ NK cells during the challenge phase, potentially primed by residual antigens from the initial sensitization. However, this needs to be further examined.

Furthermore, accumulating evidence suggests that the liver serves as a primary reservoir for NK cells possessing unique adaptive traits. Early studies demonstrated that adoptive transfer of hapten-sensitized hepatic NK cells, but not splenic NK cells, elicited recall responses to the same hapten in naïve recipient mice.^15^ Subsequent studies further demonstrated that hepatic NK cells mediate antigen-specific memory responses not only to chemical haptens but also to viral antigens such as VSV, HIV, HBV, SIV, and influenza.^16^^,^^59^^,^^61–64^ While the exact mechanism behind how NK cells mediate antigen-specific memory responses remains unclear, several markers have been implicated in defining memory NK cells, including CXCR6, CXCR3, CD49α, IL-7Rα, CD62L, KLRG1, NKG2C, and transcriptional factors like TCF1 and RORα.^15^^,^^16^^,^^18^^,^^59^^,^^65–67^ Our findings align with the importance of Ly49C/I, as we observed that this receptor, but not Ly49G, plays a critical role in mediating antigen-specific memory responses, regardless of MHC-I background. Whether these Ly49C/I-mediated responses are also mediated by hepatic NK cells requires further investigation.

Finally, we cannot discount the possibility that the reason Ly49G does not play a role in NK cell memory responses is that it is not expressed by ‘bona fide’ memory NK cells. The current known markers of memory NK cells cannot adequately discriminate between conventional NK cells and memory NK cells, making this question difficult to answer. Two possibilities exist based on whether conventional and memory NK cells are separate or derived from each other. The premise that memory NK cells derive from conventional, Ly49G-expressing NK cells, like memory T cells are derived from naïve T cells, would mean that the sharing of receptors, including Ly49C/I and Ly49G, is a result of lineage, and that the 2 receptor types have very different functions. If conventional and memory NK cells are distinct, and there is evidence to suggest that memory NK cells are a distinct ILC subset,^18^ then both types of NK cells share Ly49C/I but for different purposes: conventional NK cells use Ly49C/I for missing self-surveillance when MHC-I is downregulated, while Ly49C/I on memory NK cells forms the antigen-receptor or part of the receptor complex as supported by our earlier studies.^25^^,^^26^ In this case, Ly49G may simply not be expressed at any point in the developmental program of such memory cells. Until a memory NK cell-specific marker is identified, we cannot differentiate between these possibilities.

In conclusion, our findings show that the Ly49 receptor requirement for the formation of NK cell memory responses is not uniform. We found that Ly49C/I are critical for the formation of adaptive NK cell responses, while Ly49G, another highly expressed and conserved Ly49 receptor in mice, is dispensable for NK cell memory. Thus, Ly49C/I appears to have special properties and possibly uniquely enabling antigen-adaptive memory NK cell formation and function.

## Data Availability

The data supporting the findings of this study are available within the article. For raw data, please contact the corresponding author.
